# Book Reviews

**Published:** 1804-06-01

**Authors:** 


### CRITICAL ANALYSIS

OF THE

### RECENT PUBLICATIONS

ON THE

### DIFFERENT BRANCHES OF PHYSIC, SURGERY, AND MEDICAL PHILOSOPHY.

---

*An improved Method of treating Strictures in the Urethra; by* THOMAS WHATELY, *Member of the Royal College of Surgeons, in London.* 8vo. pp. 230. London, 1804.

( Continued from our last, pp. 475—478. )

"When the bougie has reached the anterior part of the stricture, it should rest there for a few seconds, that the caustic may begin to dissolve. It should then be pushed very gently forward, about one-eighth of an inch; after which there should be another pause for a second or two. The bougie should then be carried forward in the same gentle manner, till it has got through the stricture. The sense of feeling will generally inform the operator, when it has proceeded so far. But here we are to have recourse likewise to the notch in the bougie, as a guide; which, when the point of the instrument has fairly passed through the stricture, will generally be seen near the orifice of the urethra.

"In the greater number of cases, in which the caustic is thus applied, even in the first instance, it gives but little pain at the time of its application. A slight scalding in making water, and a trifling discharge during the first day or two, are, however, commonly produced by it. But it must be observed, that the mildness of these effects is entirely owing to the small quantity of caustic employed."

"At the expiration of seven days, the application of the caustic should be repeated in the same manner. If the first application should have enlarged the aperture of the stricture, which may be known by passing a bougie through it, of the same size as that by which the caustic was conveyed, the bougie used in the second operation should be a size larger than the one used in the first, taking care, however, that this be not too large to pass through the stricture. But if the passage shall not have been enlarged by the first process, the second must be carried on by the same sized bougie as was before used. If the patient felt no pain under the first operation, a piece of kali, a small degree larger than was used before, may be selected for the succeeding attempt. But if the first application gave pain, there should be no increase made in the quantity of caustic. At the end of seven days more the caustic should be repeated a third time. At this and all future applications of of it, the bougie, upon which it is applied, should be increased in size, in proportion as the aperture of the stricture dilates, the better to effect the dilatation. Every succeeding bougie should pass with some degree of tightness through the stricture, and be moved backwards and forwards several times, either slowly or more quickly, as the patient best can bear, till the caustic is dissolved. The operation should be repeated in this manner till the contracted part of the urethra is dilated, if possible, to the natural size, which is generally practicable in recent strictures. In those of longer standing, we should make the attempt, and carry the dilatation as far as it can be done with safety. We are, however, on no account to increase the quantity of caustic, as we increase the size of the bougie.

" On account of the extreme activity of this remedy, and the certainty with which it destroys the organization of the part it touches, it is necessary to fix upon a maximum or determinate quantity, which we should not venture in any case to exceed. Without this precaution, great mischief may result from its application; as some patients have so little irritability, as to be able to bear much larger quantities of it, without any sensible inconvenience, than it would be prudent to use. On this account, as well as from a wish that the remedy should act only on the surface of the stricture, without destroying its substance, I do not in any case apply more of the kali purum at a time than a piece about the size of a common pin's head; and even this quantity cannot be borne where the habit is very irritable. It would be difficult to weigh such small portions of the article. In order, therefore, to convey a clear idea of the different quantities to be used, I shall here represent them by three dots of different sizes, thus, • • . and to give further assistance on this important point, I find that twelve bits of the largest size weigh one grain."

The succeeding chapter contains Mr. W's method of applying *lunar caustic* to strictures, as recommended in his former work; for he by no means wishes this remedy to be superseded by the new one, as different cases require different treatment.

" There are, (says Mr. W.) as has been before stated, some cases in which the contraction is so irregular, and its aperture so untowardly situate, that a bougie cannot readily, if at all, be passed into it; other cases have likewise been described, in which it is impossible to pass a bougie through the strictures. If, in the former of these cases, a bougie, furnished with the kali, cannot be passed *into* the stricture, or if it get through the stricture, and yet do not destroy the irregularity, and it becomes necessary for this purpose to apply a caustic at the extremity of a bougie to the anterior part of the contraction, I should certainly prefer the lunar caustic to the kali purum. In cases of the latter description, the lunar caustic, for the reasons already assigned, ought always to be fairly tried in the same way, previous to the use of the kali purum.

" The lunar caustic should be used in every case, in a determinate nate quantity, upon the extremity, and partly upon the shoulders of a common bougie, which has previously had the curvature of the urethra given to it. The following is an improved method of arming a bougie with this caustic. Take a given quantity of powdered lunar caustic, e. g. the twelfth part of a grain, put it upon a bit of flat glass, then take a very small quantity of thick mucilage of gum arabic upon the point of a pen knife, (about the quantity of a large pin's head to a quarter of a grain of caustic is sufficient) mix the caustic and mucilage together. If the quantity of mucilage be nicely adapted to that of the caustic, it will almost immediately form a paste, which may be taken up from the glass on the point of a knife. In about half a minute this will become so stiff, that it may be rolled into a pill, without adhering to the fingers. In this state it should be put immediately upon the end and shoulders of the bougie, and without delay so moulded upon it, as to become perfectly smooth, like the extremity of the bougie. The operator's finger and thumb will mould the paste, and his thumb nail will smooth its surface, or it may be polished by gently rubbing it on the glass. But this must be done expeditiously, otherwise the paste will become so hard as to be incapable of being thus moulded."

"The bougie may likewise be prepared in the following manner: The liquid paste of caustic and mucilage, when first mixed, may be immediately put upon the extremity of the bougie from the point of the knife, and, as it hardens, may be coated upon it, partly by one of the fore fingers, and partly by the knife. This paste hardens so quickly, that by the following day it will be too firmly fixed on the bougie to be easily separated from it.

"When a bougie, thus armed, is applied to either of the kinds of stricture I have just mentioned, it should be of as large a size as the urethra will admit. When it arrives at the strictured part, it should be pressed against it with a moderate degree of force, till the caustic is dissolved, which it will be in a minute or two.

"The quantity of caustic employed in the first operation, should not in any case exceed the sixteenth part of a grain. At the second and third operation, the twelfth part of a grain may be used, if the quantity first employed gave but little pain. If, after this, no progress is made in opening the stricture, the quantity of caustic may be gradually increased to the eighth, the sixth, or if the patient bear the preceding quantity very well, even to the fourth of a grain at each application. But the increase should be made with a very cautious hand, and in no case ought we to exceed the quantity last mentioned. In those who are young and healthy, it may sometimes in these cases be repeated to advantage every five days; in other cases, however, a week, or even less frequently, will be sufficient. In making an addition to the quantity before used, we should attentively regard the effect produced by preceding applications; should these have produced pain and irritation, with a frequent desire to make water; or hæmorrhage, even of the slightest kind; we must not even repeat the caustic, much less less increase its quantity, till these effects have entirely ceased. As a proof of the necessity of keeping an attentive eye on the effect of former applications, I may inform the reader, that I have seen an instance, where only the twenty-fourth part of a grain of this caustic produced great irritation."

The remaining part of the volume contains observations on the danger, suffering, and consequent inconvenience of Mr. Home's method of applying caustic to strictures; to which succeed a competent number of cases to illustrate the use of both remedies.

There is also an APPENDIX, on a peculiar affection of the bladder in elderly people.

In our opinion, all sufferers from strictures in the urethra, as well as surgeons in general, are much indebted to Mr. Whately for the important improvements he has made in this branch of the profession.

---

*Three Letters on Medical Subjects: addressed to the Rev. Gilbert Ford, Ormskirk, Lancashire. By* JOHN FORD M. D. *Chester.* London, pp. 55. 1803.

THE first and principal part of this little publication is no more a subject for medical criticism than the advertisements of the fashionable empirics of the day. It will only excite some surprize, that a regular physician should depart from the line of professional respectability, in thus advertising a secret medicine prepared under his own inspection, and taking pains to satisfy the world that the secret will remain in the family, unimpaired, after the death of the inventor.

The next subject is, remarks on the use of granulated tin as a vermifuge, and as a medicine of general activity in relieving the intestines from indurated fæces long retained. Besides this well-known power of tin filings, the author attributes to it, when used in large doses, a peculiar sedative operation in checking hæmoptysis, and in quieting various powerful nervous impressions on the mind, without being attended with the same inconveniencies that follow the use of opiates. With this view Dr. F. particularly advises the tin to be employed in the latter stages of pregnancy, when the mind is often peculiarly anxious and irritable, and when at the same time, a state of costiveness ought to be carefully avoided. A sedative power has, we believe, never been before attributed to this remedy; if well founded, possibly it may be owing to the lead with which the tin in common use is generally alloyed in a small proportion, and which may have a sensible operation in the large doses employed by the author.

In an appendix is added the following process for preparing the *hydrargyrus cum magnesia* or *mercurius alkalizatus.*

"Take of purified quicksilver, the finest and cleanest manna, of each one ounce. Rub them well together in a stone mortar, until the mercurial globules entirely disappear, which will be in a few minutes; minutes, adding a few drops of water if necessary, and afterwards as much more as will reduce the mixture to the consistence of a thick syrup or treacle. With this, incorporate, by trituration, one drachm of magnesia, and add a pint of tepid water.—After the mercury has subsided, pour off the clear liquor, and add another pint of tepid water as before; and decant it off after the subsidence of the powder: To this powder add three drachms of magnesia, and as much water as will reduce it to the consistence of an electuary.—After a little trituration, put the mass upon filtering paper, and dry it with a gentle heat, breaking it now and then to expedite the drying. When it is *thoroughly* dried, add two drachms of gum arabic in powder, and mix them well in a stone mortar."

This method of using manna for the extinction of the mercury, and afterwards washing it out, leaving the metal combined with the earth, appears to us a real improvement, and worthy of attention.

---

*Elements of Materia Medica and Pharmacy. By* J. Murray, *Lecturer on Chemistry, and on Materia Medica and Pharmacy.* 2 vol. 8vo. pp. 746. Edinburgh, 1804.

This work is divided into three parts: the first treats of pharmaceutic chemistry, or of those parts of general chemistry that relate to pharmacy; the second contains a classification of all the subjects of the materia medica, with a summary view of these properties and the chief modes of combination in which they are employed; the third part is the pharmacopœia, or rules for the preparation of the different medicines, chiefly from the last edition given by the Edinburgh College, collated with the London Pharmacopœia, together with remarks on the rationale of each prescription where any thing occurs worthy of notice.

We shall give a short view of each section.

The part devoted to pharmaceutical chemistry begins with a slight sketch of the chemical elements, some of which can only be introduced for the sake of uniformity, as they have no connection with pharmacy. The author, with propriety, enlarges on the analysis of vegetable matter as more peculiarly relating to the preparation of medicines. He very justly enforces the important distinction between the proximate and the elementary principles of vegetables, and shews that the business of the pharmaceutical chemist altogether relates to the former, not the latter.

The proximate principles being described, the methods of separating and preparing them for use, and the re-agents necessary for this purpose, are thus enumerated.

"From this enumeration of the proximate principles of vegetables, we may perceive the reasons for those pharmaceutic processes to which plants are usually subjected.

"By infusion in water, we impregnate the fluid with the gum, sugar, extract, tannin, saline substances, part of the essential oil, and part also of the resinous principle. The aroma of the plant is

 generally generally first taken up: by longer infusion the water is loaded with the colouring astringent and gummy parts: these are also most abundantly dissolved when the temperature is high. Hence an infusion differs according as the water has stood, longer or shorter, on the materials, and according as it has been promoted or not by heat. An infusion made in the cold is in general more grateful, while one made with heat, or by keeping the fluid long upon the materials, is more strongly impregnated with active matter.

" By decoction or boiling, the solvent power of the water is still farther increased; and hence the liquor always appears darker coloured, and is, in fact, more loaded with the principles of the vegetable which it can hold dissolved. The volatile parts, however, particularly the essential oil, are entirely dissipated; and therefore it is an improper process for those vegetables whose virtues depend, wholly or partially, on these parts. Even the fixed principles of vegetables, at least some of them, are injured by long decoction. The extractive matter, for instance, gradually absorbs oxygen from the atmosphere, and is converted into a substance nearly insipid and inert. Opium, Peruvian bark, and many other vegetables, are injured in this manner by decoction, especially if the atmospheric air is freely admitted; and these two circumstances, the dissipation of the volatile matter, and the oxygenation of the extractive, considerably limit the application of this process. It is still used, however, with advantage, to extract the mucilaginous parts of vegetables, their bitterness, and several other of their peculiar qualities.

" Alkohol may be applied to vegetables to extract those principles which are not soluble in water. It dissolves entirely their essential oil, camphor, and resin; and as these are often the parts on which the virtues of vegetables depend, these solutions, or tinctures, as they are termed, are often active preparations.

" Equal parts of alkohol and water, in general, extract still more completely the active matter of plants, as we thus obtain a solution of all those substances which are separately soluble in either of these fluids.

" When by the action of one or both of these fluids, we obtain a solution of the active principles of a vegetable, the solution may be evaporated to the consistence of a thick tenacious mass. This forms what is termed an extract: it is termed an aqueous extract when obtained from the aqueous infusion or decoction of a plant, and spirituous when alkohol has been the solvent. The design of this preparation is to obtain the active matter of the vegetable in a small bulk, and in such a state that it may be preserved a long time without suffering any alteration. It is evident, that it is a process which can be properly applied to such plants only as have their virtues dependant on some of their fixed principles, and even these are often injured by the heat employed, and the free access of the atmospheric air.

" Distillation is another process applied to vegetable substances, by by which we obtain some of their active principles, particularly their essential oil. If the vegetable matter be heated along with the water, the oil is volatilized, alongst with the aqueous vapour: it separates from the water on being allowed to remain at rest; a part of it, however, is also dissolved, and communicates to the water a considerable degree of flavour, and often also of pungency. This forms what are termed distilled waters. If alkohol be used instead of water, the essential oil is completely dissolved in it, and we thus obtain what are termed distilled spirits."

The oxygenation of the solutions of those substances which liquid menstrua are able to extract from vegetables, has been supposed by several chemists to impair their medicinal virtues. Mr. M. is of the same opinion, and he gives the example of the decoction of cinchona in illustration.

" From an accurate analysis of it, (the cinchona) it has been proved that seven parts out of eight of it consist of woody fibre, or of a matter inert and insoluble, which cannot act on the system, and which affects the stomach only by its weight and insolubility. The remaining eighth part is that in which the activity of the medicine resides: it is therefore evident that if this be extracted, without injuring its activity, the medicine could be exhibited with much more advantage. This is in part accomplished by the preparations of it that have been mentioned; but even these do not convey it in all its force. If one ounce of the bark be infused or boiled in a certain quantity of water, the infusion or decoction is not nearly equal in efficacy to the whole quantity of bark operated on. It is therefore evident that during either of these operations, the active matter of the bark has not been entirely extracted, or has suffered some change. And here chemistry lends her assistance, and still farther elucidates the peculiar nature of this substance, and the changes produced in it by these processes. It has been proved by experiments, that the matter on which the power of the bark depends, has a strong attraction for oxygen at a temperature moderately increased; that during the infusion, and particularly during the decoction of that drug, this active matter absorbs oxygen from the atmosphere, and is converted into a substance insipid, and inert. This leads to the improvement of the preparations of this medicine; and experiments instituted for the purpose have accordingly proved, that, while by long boiling the virtues of the bark are nearly totally destroyed, they are fully extracted by a few minutes decoction in covered vessels. The same investigations have pointed out the nature of the action of some other substances on bark, formerly not well understood. Thus, it has been found by experience, that the alkalies, and more particularly magnesia, enable water to extract the virtues of bark, more completely by infusion—a circumstance elucidated by the fact since discovered, that the extractive matter of the bark, to which its activity is owing, combines with facility with these substances, and forms soluble compounds."

The

The author's classification of medicines is in some degree different from his predecessors, principally indeed by following the simplifying system of Brown with very scrupulous accuracy. The author's table of classification is the following.

| | | |
|---|---|---|
| " A. GENERAL STIMULANTS. | | |
| | a. Diffusible. | Narcotics. |
| | | Antispasmodics. |
| | b. Permanent. | Tonics. |
| | | Astringents. |
| B. LOCAL STIMULANTS. | | Emetics. |
| | | Cathartics. |
| | | Emmenagogues. |
| | | Diuretics. |
| | | Diaphoretics. |
| | | Expectorants. |
| | | Sialagogues. |
| | | Errhines. |
| | | Epispastics. |
| C. CHEMICAL REMEDIES. | | Refrigerants. |
| | | Antiacids. |
| | | Lithontriptics. |
| | | Escharotics. |
| D. MECHANICAL REMEDIES. | | Anthelmintics. |
| | | Demulcents. |
| | | Diluents. |
| | | Emollients." |

All that has been said or can be said on the subject of stimulant and sedative, in the present state of human knowledge, has long been exhausted; we shall therefore abstain from entering into this controversy, but we must make a single remark on the mode in which the author introduces this arrangement to his readers. He observes, that " the systems of classification of the articles of the materia medica, which are founded on their sensible qualities, their chemical compositions and properties, or their characters as objects of natural history, are extremely defective. They associate substances, which, as medicines, have little resemblance, and separate others which are intimately connected.

" As the study of the materia medica is merely the study of the medicinal properties of certain substances, it is evident that the method of arranging them, as they agree in producing effects on the living system, is the one best calculated to fulfil all its objects.

" The foundation of the classes being similarity of operation, those substances are arranged together which have the closest resemblance in medicinal power, and although, when the extremes of the classes are considered, substances may sometimes be found associated which appear to be little connected, yet this can never be so much so as in the other systems of classification, and the connection nection, though apparently remote, may always by slight gradations be traced."

Classification is so good a thing in itself that an indifferent one is perhaps better than none at all, for experience will sooner or later correct the errors of defective system; but if similarity of operation is to be the basis of a classification of materia medica, we know not where in any of the other systems will be found a more palpable violation of this principle than in the present, which includes, under the same head, alcohol, tobacco, and digitalis.

The description of each individual article of materia medica is brief, accurate, and as comprehensive as is compatible with the limits the author has allowed himself. The following will serve as a specimen:

"Angustura is a bark imported within these few years from the Spanish West Indies, the botanical characters of the tree producing it being unknown. It is in flat pieces, externally grey and wrinkled; internally, of a yellowish brown, and smooth; has little flavour; taste, bitter and slightly aromatic. Water, assisted by heat, takes up the greater part of its active matter, which does not seem to be injured even by decoction. Alkohol dissolves its bitter and aromatic parts, but precipitates the extractive matter dissolved by water. Proof spirit is its most proper menstruum. By distillation, it affords a small quantity of essential oil. The powdered bark, triturated with lime or pot-ash, and water, gives a smell of ammonia.

"Angustura is a powerful antiseptic. It was originally introduced in the West Indies as a remedy in fevers, equal or even superior to the Peruvian bark. In this country it has been principally used in obstinate diarrhœa, and in chronic dysentery, and with advantage. Its dose is from ten to twenty grains of the powder, or one drachm in infusion or decoction. Its tincture with proof spirit in a dose of one or two drachms has been used in dyspepsia."

Among the remedies supposed to act merely chemically, and whose operation is not essentially connected with the state of the living fibre, the author includes the class of refrigerants. Their modus operandi has long been a matter of dispute; the following chemical explanation of it is here given.

"Keeping in view the very inconsiderable action of these remedies, it may perhaps be possible, from the consideration of the mode in which animal temperature is generated, to point out how their trivial refrigerant effects may be produced.

"It has been sufficiently established, that the consumption of oxygen in the lungs is materially influenced by the nature of the ingesta received into the stomach; that it is increased by animal food and spirituous liquors, and in general by whatever substances contain a comparatively small quantity of oxygen in their composition. But the superior temperature of animals is derived from the consumption of oxygen gas by respiration. An increase of that consumption must necessarily, therefore, occasion a greater evolution tion of caloric in the system, and of course an increase of temperature, while a diminution in the consumption of oxygen must have an opposite effect. If, therefore, when the temperature of the body is morbidly increased, substances be introduced into the stomach, containing a large proportion of oxygen, especially in a state of loose combination, and capable of being assimilated by the digestive powers, the nutritious matter received into the blood must contain a larger proportion of oxygen than usual; less of that principle will be consumed in the lungs, by which means less caloric being evolved, the temperature of the body must be reduced; and this operating as a reduction of stimulus, will diminish the number and force of the contractions of the heart.

"It might be supposed that any effect of this kind must be trivial, and it actually is so. It is, as Cullen has remarked, not very evident to our senses, nor easily subjected to experiment, and is found only in consequence of frequent repetitions."

The contents of the third part of this work have been mentioned to be a translation of the new Edinburgh Pharmacopœia, with an addition of those preparations of the London College that are peculiar, or differ essentially from the former. To some of the metallic and saline preparations, suitable remarks are added on the rationale of the operation. Under the *pulvis antimonialis*, Mr. Chenevix's new method is given; but the author adds, (what we believe to be perfectly well founded) that neither this nor the antimonial powder of the College is precisely the same as the celebrated empiric preparation which they profess to imitate.

The dangerous nomenclature now adopted by the Edinburgh College for calomel and corrosive sublimate, is here noticed.—Mr. M. prefers the old distinctions of *corrosivus* & *mitis*.

The first appendix to these volumes contains a very short view of the medical history of the gasses, of electricity, and Galvanism.

In the second appendix a few remarks are given on the method of composing medical prescriptions, with a table of doses.

"The following are the principal circumstances to be attended to in forming a prescription.

"1*st*, Simplicity should be attained, as far as is consistent with the objects of the prescription. Nothing ought to enter into the composition which does not add to its virtue, render it less ungrateful, give it a convenient form, or which is not necessary to conceal any particular ingredient; and, in general, the practice of accumulating a number of articles in one prescription is to be avoided.

"2*dly*, Substances, it is evident, ought not to be mixed together, which are capable of entering into chemical combination, or of decomposing each other, unless it be with a view of obtaining the product of the combination, or decomposition, as a remedy.

"3*dly*, Those mixtures are also to be avoided, in which one medicine, by its peculiar action on the stomach or general system,

 modifies modifies and changes the action usually exerted by another, unless where the object is to obtain the effects of that modified operation.

"*4thly*, The error of contra-indication is to be guarded against, or those medicines ought not to be combined, the virtues of which are not merely different, but are, in some measure, opposed to each other.

"*5thly*, The ingredients which are to be mixed, must be such as will mix properly together, so that the form in which the remedy is designed to be exhibited, may be easily obtained and preserved.

"*Lastly*, The form under which a medicine is prescribed, must be adapted to certain circumstances; principally to the nature of the disease, the nature of the remedy itself, and, as far as may be possible, to the taste of the patient.

An experienced apothecary might fill up these outlines to great advantage: as they now stand, they scarcely deserve a place.

Tables and an Index conclude the work.

---

*An Essay, Medical, Philosophical, and Chemical, on Drunkenness and its Effects on the human Body; by* THOMAS TROTTER, M.D. &c. &c. 8vo. pp. 203. London, 1804.

THIS Essay is a translation, much enlarged, of Dr. T's Thesis, which he defended for his degree at Edinburgh, in 1788. It continued very popular among the students for several years, on account of the novelty of the subject, its own merit, and particularly the concise and pointed encomium of Dr. Cullen, who was his public Examiner at the Graduation. It is the custom in this truly liberal University for the Professor who examines the candidate in public, to address him in a short speech on the subject of his Thesis, in order to take off the embarrassment that a young man must feel on commencing the defence of it in Latin, before a large audience, a majority of which are good judges of what they hear, and this too against an able and eloquent Professor, who speaks Latin as fluently as English. Dr. Cullen commenced his address on this occasion in the following words, which did the candidate no less honour than his degree, "Hanc de ebrietate dissertationem non ebrius scripsisti."

Dr. T. introduces his subject by a general statement of its importance, and the neglect it has experienced among medical writers.

"Mankind, ever in pursuit of pleasure, have reluctantly admitted into the catalogue of their diseases, those evils which were the immediate offspring of their luxuries. Such a reserve is indeed natural to the human mind; for of all deviations from the paths of duty, there are none that so forcibly impeach their pretensions to the character of rational beings as the inordinate use of spirituous liquors. Hence, in the writings of medicine, we find drunkenness only cursorily mentioned among the powers that injure health, while the mode of action is entirely neglected and left unexplained.

This

This is the more to be wondered at, as the state of ebriety itself exhibits some of the most curious and interesting phænomena that are to be met with in the history of animated nature. The potent stimulus of vinous spirit, as if by magical influence, so disturbs or operates on the animal functions, that new affections of mind, latent or unknown before, are produced; and the drunkard appears to act the part of a man of deranged intellect, and altogether foreign to the usual tenor of his sober reflections.

"But a long train of the most dangerous diseases are the certain consequence of habitual intoxication: the body and mind equally suffer. Sudden death, apoplexy, palsy, dropsy, madness, and a hideous list of mental disquietudes and nervous failings, prey upon the shattered frame of the inebriate, and prove fatal in the end. These sufficiently point out the subject as highly important in a medical view, and worthy of the nicest investigation. But as I have not any precursor in my labours, nor example in the records of physic, to direct my steps, I shall need the less apology for the manner I mean to pursue; and must claim indulgence where I appear singular in my method.

"In order to treat my subject philosophically, and for the sake of method, I propose dividing it into the following heads, viz.

"1st. Definition of drunkenness.

"2d. The phænomena, or symptoms of drunkenness.

"3d. In what manner vinous spirit affects the living body.

"4th. The catalogue of diseases induced by drunkenness. And,

"5th. The method of correcting the habit of drunkenness, and of treating the drunken paroxysm.

"Under these heads I shall occasionally introduce such practical remarks as may arise out of the subject, but which are too desultory for methodical arrangement.

When Dr. T. comes to treat of the way in which the health is undermined by intoxication, he is naturally led to consider the operation of *narcotics* in general upon the human body; and here he cannot avoid the question, so much agitated, respecting the *stimulant* and *sedative* powers of these substances. He defends, at considerable length, the opinion which seems to be most generally received, viz. That these poisons *may* be always so administered as to be *stimulant* in the first instance, and so as not to be *perceptibly* sedative in their *secondary* effects; they may, on the contrary, be so administered, that the stimulant effect may not be perceptible, and the sedative one truly alarming, if taken in immoderate doses. That in moderate doses they are all stimulant.

Among the most remarkable effects of ebriety is the power it gives of resisting *cold* and *contagion*.

"The drunkard is found, in the first stage of the paroxysm, to resist the operation of cold. No stronger proofs of this need be adduced than what are daily observed among our seamen in the naval sea-ports. These men are permitted to come on shore to recreate themselves; but, from a thoughtlessness of disposition, and the the cunning address of their landlords, they drink till the last shilling is spent; they are then thrust out of the door, and left to pass the night on the pavement. It is surprising how they should escape death on such occasions; for I have known many of them who have slept on the street the greatest part of the night in the severest weather. Nothing but that hardiness of constitution peculiar to the British seamen, which braves every danger, could survive such extremes of cold.

"The following fact is a strong instance of the inebriate resisting cold. A miller, very much intoxicated, returning from market late at night, while it snowed and froze hard, missed his way, and fell down a steep bank into the mill-dam. By the fright and sudden immersion, he became so far sensible as to recollect where he was. He then thought the surest way home would be to follow the stream, which would take him within pistol-shot of his own door. Instead, however, of taking that course he waded against the current, without knowing it, till his passage was opposed by a wooden bridge. This bridge he knew; and though he felt some disappointment, he still thought his best way was to follow the stream, for the banks were steep and difficult to climb. He now found himself in a comfortable glow; turned about, and arrived at his own house at midnight, perfectly sober, after having been nearly two hours in the water, and often up to the breech. He went immediately to bed, and rose in perfect health.—As the senses were recovered at the time he got home, it is probable he could not have resisted the cold much longer. This instance tends to confirm a common observation, that sudden immersion in cold water puts a speedy end to intoxication."

[To be continued.]

